# Supplementary material for: Potential Pharmacokinetic Drug–Drug Interaction Between Harmine, a Cholinesterase Inhibitor, and Memantine, a Non-Competitive N-Methyl-d-Aspartate Receptor Antagonist
Source: Molecules. 2019 Apr 11;24(7):1430. doi: 10.3390/molecules24071430 (PMC6479946; doi:10.3390/molecules24071430)
Supplement: Supplementary file 1 [file molecules-24-01430-s001.pdf]

Supplementary materials

Potential Pharmacokinetic Drug-Drug Interaction Between Harmine, a Cholinesterase Inhibitor, and Memantine, a Non-Competitive N-Methyl-D-Aspartate Receptor Antagonist

Yunpeng Zhang , Shuping Li [1], Youxu Wang [1], Gang Deng [1], Ning Cao [1], Chao Wu [1], Wenzheng Ding [1], Yuwen Wang [1], Xuemei Cheng [1, 2] and Changhong Wang [1, 2,*]

[1] The MOE Key Laboratory for Standardization of Chinese Medicines and The SATCM Key Laboratory for New Resources and Quality Evaluation of Chinese Medicine, Institute of Chinese Materia Medica, Shanghai University of Traditional Chinese Medicine, 1200 Cailun Rood, Shanghai 201203, China; zhangyp1028@163.com (Y.Z.); lishupinghappy@163.com (S.L.); wyxzd1314@163.com (Y.W.); 18930900232@163.com (G.D.); 18616024782@163.com (N.C.); vera105370@163.com (C.W.); 18817385708@163.com (W.D.); wangyuwen92@163.com (Y.W.); chengxuemei1963@163.com (X.C.)

[2] Shanghai R&D Centre for Standardization of Chinese Medicines, 1200 Cailun Rood, Shanghai 201203, China

* Correspondence: wchcxm@hotmail.com or wchcxm@shutcm.edu.cn; Tel: +86-021-5132-2511; Fax: +86-021-5132-2519

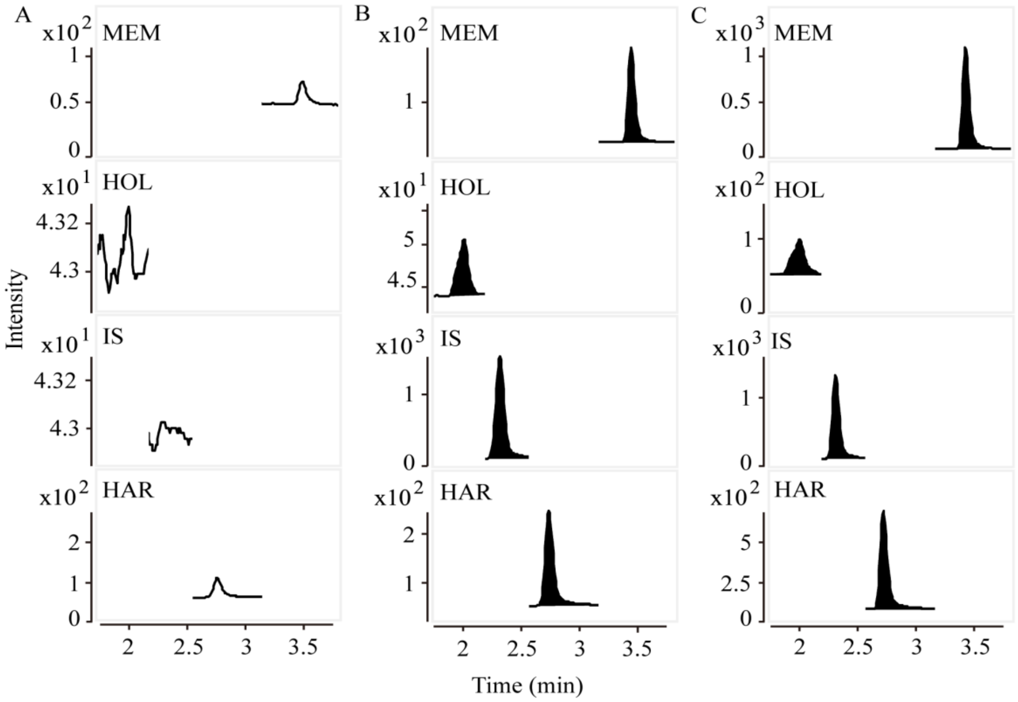

Supplementary materials Figure S1. Representative MRM chromatograms of HAR, MEM, HOL and IS in rat plasma: (A) a blank plasma; (B) a blank sample spiked with the analytes (with LLOQ) and IS (40.0 ng/ml); and (C) a plasma sample (2.0 min) from a rat after the co-administration of medium dose (40.0 mg/kg) HAR with 5.0 mg/kg MEM.

Table S1. MS/MS conditions for multiple reaction monitoring of MEM, HAR, HOL and IS.

Analytes	Precursor Ion	Product Ion	Dwell	Fragmentor	Collision Energy	Cell Accelerator Voltage	Polarity
MEM	180.2	163.1	120	90	16	1	Positive
HAR	213.2	170.0	120	125	35	1	Positive
HOL	199.2	131.1	120	155	36	1	Positive
IS	199.2	144.1	120	170	45	1	Positive

Table S2. LLOD, LLOQ and representative calibration curves of each standard substance (n=5).

Analytes	LLOD (ng/mL)	LLOQ (ng/mL)	Linear range (ng/mL)	Slope	Intercept	R^2
MEM	0.10	0.40	0.40-400	0.550	0.00638	0.997
HAR	0.10	0.40	0.40-400	3.23	0.0294	0.998
HOL	0.10	0.40	0.40-400	4.05	-0.00185	0.998

Table S3. Summary of within-run and between-run precision for the UPLC-MS/MS method (n=6).

Analytes	Nominal level (ng/mL)	Within-run precision			Between-run precision		
		Mean±SD	CV (%)	RE (%)	Mean±SD	CV (%)	RE (%)
MEM	0.40(LLOQ)	0.426±0.0184	4.32	6.59	0.424±0.00272	0.640	6.15
	1.00(QCL)	1.09±0.0319	2.93	8.87	1.06±0.0241	2.27	6.24
	100(QCM)	102±2.69	2.63	2.08	101±3.71	3.67	1.01
	300(QCH)	311±5.79	1.86	3.79	303±12.1	4.00	1.06
	400(ULOQ)	414±26.8	6.48	3.41	401±13.6	3.40	0.147
HAR	0.40(LLOQ)	0.471±0.0153	3.26	17.6	0.441±0.0265	6.02	10.1
	1.0(QCL)	1.10±0.0408	3.71	9.90	1.08±0.0267	2.47	8.18
	100(QCM)	106±3.99	3.75	6.45	103±4.66	4.54	2.67
	300(QCH)	302±8.02	2.66	0.606	298±8.49	2.85	-0.750
	400(ULOQ)	398±29.8	7.50	-0.530	396±5.08	1.28	-1.02
HOL	0.40(LLOQ)	0.449±0.0398	8.86	12.3	0.434±0.0170	3.92	8.59
	1.0(QCL)	1.08±0.0621	5.73	8.39	1.07±0.0192	1.80	6.68
	100(QCM)	97.7±2.54	2.60	-2.30	98.7±3.95	4.00	-1.29
	300(QCH)	276±5.08	1.84	-8.15	286±13.8	4.83	-4.80
	400(ULOQ)	366±8.68	2.37	-8.59	377±11.4	3.02	-5.83

Table S4. Summary of extraction yield and matrix effect for the UPLC-MS/MS method (n=6).

Analytes	Add conc. (ng/mL)	Measured conc. (ng/mL, mean±SD)	Matrix effect (%, mean±SD)	Recovery (%, mean±SD)
MEM	1	1.05 ± 0.0440	96.4±3.20	105±4.40
	100	104 ± 6.34	102±7.50	104±6.34
	300	298 ± 7.09	98.2±1.96	99.5±2.36
HAR	1	1.05±0.0321	98.0±4.63	105±3.21
	100	102±5.63	101±6.99	102±5.63
	300	299±4.48	99.2±1.77	99.6±1.49
HOL	1	1.06±0.0382	102±2.17	106±3.82
	100	102±3.94	102±5.03	102±3.94
	300	292±6.06	98.7±2.74	97.2±2.02
IS	40	41.1±0.265	99.0±1.68	103±0.663

Table S5. Stability of each standard substance in rat plasma under different storage conditions (n=5).

Conditions	Nominal levels	MEM		HAR		HOL	
		Mean±SD	CV%	Mean±SD	CV%	Mean±SD	CV%
AT	QCL	1.06±0.0138	1.31	1.10±0.0236	2.15	1.07±0.0111	1.04
	QCM	104±3.90	3.75	104±3.90	3.74	103±2.77	2.69
	QCH	309±4.65	1.50	303±5.16	1.70	301±9.79	3.25
4˚C	QCL	1.09±0.0319	2.93	1.10±0.0408	3.71	1.08±0.0621	5.73
	QCM	102±2.69	2.63	106±3.99	3.75	97.7±2.54	2.60
	QCH	311±5.79	1.86	302±8.02	2.66	276±5.08	1.84
-20˚C	QCL	1.04±0.0552	5.30	1.05±0.0466	4.43	1.05±0.0446	4.27
	QCM	96.9±4.32	4.45	97.5±6.51	6.68	95.4±3.80	3.98
	QCH	289±16.5	5.71	288±14.9	5.17	270±11.1	3.97
Freeze/thaw	QCL	1.05±0.0440	4.20	1.05±0.0321	3.04	1.06±0.0382	3.58
	QCM	104±6.34	6.09	102±5.63	5.51	12.0±3.94	3.87
	QCH	298±7.09	2.38	299±4.48	1.50	292±6.06	2.08
